# Inhibition of lung tumor growth in nude mice by siRNA^CD31^ targeting PECAM-1

**DOI:** 10.3892/ol.2014.2091

**Published:** 2014-04-25

**Authors:** JIN-SHENG OUYANG, YU-PING LI, CHENG-SHUI CHEN, JUN-JIE CHEN, TONG-KE CHEN, CHANG CAI, LI YANG

**Affiliations:** Department of Respiratory Medicine, The First Affiliated Hospital of Wenzhou Medical University, Wenzhou, Zhejiang 32500, P.R. China

**Keywords:** small interfering RNA, platelet endothelial adhesion molecule, vascular endothelial growth factor, carcinoma, tumor microenvironment

## Abstract

Small interfering RNA (siRNA) provides a promising therapeutic approach in the silencing of disease-causing genes. In the present study, the use of 2′-O-methyl-modified siRNA-cluster of differentiation 31 (siRNA^CD31^), with cationic liposome RNA interference (RNAi)-mate as a carrier, effectively silenced the platelet endothelial cell molecule 1 (PECAM-1) gene of murine hemangioendothelioma cells *in vitro. In vivo*, 2′-O-methyl-modified siRNA^CD31^ carried by RNAi-mate was successfully delivered, targeting the PECAM-1 gene in the vasculature of nude mouse lung carcinoma xenografts. The growth of the lung carcinoma xenografts was inhibited by the 2′-O-methyl-modified siRNA^CD31^ and RNAi-mate complexes, and the expression of the PECAM-1 protein was downregulated, with a simultaneous decrease in vascular endothelial growth factor (VEGF) protein in the lung carcinoma xenografts. 2′-O-methyl-modified siRNA^CD31^-RNAi-mate complexes may provide a potential therapeutic strategy in lung carcinoma treatment. The effect of PECAM-1 on VEGF expression may possibly be attributed to the function of PECAM-1 signal transduction.

## Introduction

Lung cancer is a leading cause of mortality globally, with the most frequent type being adenocarcinoma. Although platinum-based traditional chemotherapy is currently the first-line therapy for advanced lung cancer, due to its clinical benefits, its use is limited due to significant associated toxicities. In an effort to overcome these limitations, targeted therapies are currently an area of research focus due to our progressive understanding of tumor molecular biology and the tumor microenvironment (TME), including medications targeting the epidermal growth factor receptor, such as gefitinib (1,2) and erlotinib (1,3), and those targeting the vascular endothelial growth factor (VEGF) signaling pathways, such as bevacizumab (4). However, only a small proportion of specific patients benefit from the current targeting agents, with inevitable resistance. The identification of alternative promising molecular targets would be a rational consideration for individual patients with lung cancer.

Tumor growth and invasion is closely associated with the TME. The main components of TME angiogenic endothelial cells are regulated by various bio-mediators, including platelet endothelial cell molecule 1 [PECAM-1; namely cluster of differentiation 31 (CD31)] (5,6) and VEGF (7–9). PECAM-1 is a biomarker of endothelial cells (10–12). Experimental studies have indicated that PECAM-1 regulates endothelial cell motility and angiogenesis (13) and is a potential target on TME endothelial cells (14,15). Although it has been shown that the vascular inhibitor that targets the VEGF of the TME can be used an efficacious therapy (4,9,16–19), it remains uncertain whether PECAM-1 could be used as an angiogenic inhibitor on the TME. In addition, the delivery system targeting PECAM-1 *in vivo* requires further exploration.

RNA interference (RNAi) technology shows considerable promise as a nucleic acid-based therapy (20). Small interfering RNA (siRNA) consists of 19- to 23-nucleotide double-stranded RNA duplexes via the formation of an RNA-induced silencing complex (RISC). RISCs specifically identify homologous gene mRNA and induce sequence-specific mRNA degradation leading to silencing of target gene expression. The performance of siRNA-targeted therapy requires a suitable and effective carrier delivery system. Cationic liposomes have been used as effective siRNA carriers *in vitro* and *in vivo* (21,22). Achieving systemic RNAi *in vivo* requires that the siRNA possesses the properties of stability, cellular delivery and tissue bioavailability. Aside from siRNA alone (naked), 2′-O-methyl-modified siRNA^CD31^ has the strongest resistance towards degradation by exo- and endonucleases in the serum and tissue homogenates (20,23), leading to more effective therapeutic RNAi *in vivo*.

With respect to previous discussions regarding siRNA delivery systems, the use of 2′-O-methyl-modified siRNA^CD31,^ with cationic liposomes as carriers, would be an attractive candidate technology for systemic delivery of PECAM-1 *in vivo* (20,22,23). In the present study, the effects of the systemic delivery of siRNA^CD31^ on the growth of lung adenocarcinoma xenografts were investigated with the application of 2′-O-methyl-modified siRNA^CD31^-cationic liposome complexes to silence PECAM-1.

## Materials and methods

### siRNA and RNAi-mate

The 2′-O-methyl-modified siRNA^CD31^ molecules used in the present study are described in Table I. siRNA^CD31^, 3′-fluorescein amidite (FAM) fluorescence-labeled siRNA^CD31^ (siRNA^CD31^-FAM; described in Table I), stable negative control RNA (SNC; described in Table I) and RNAi-mate were all synthesized by GenePharma Co., Ltd. (Shanghai, China). The primers of PECAM-1 mRNA for reverse transcription polymerase chain reaction (RT-PCR) were also synthesized by GenePharma Co., Ltd. (Table II).

### Cell lines and cell treatment

EOMA cells were obtained from the American Type Culture Collection (Manassas, VA, USA) and grown in endothelial growth MED-0002 media (PriCells Biomedical Technology Co., Ltd, Wuhan, China) containing 10% fetal bovine serum (Gibco, Invitrogen Life Technologies, Carlsbad, CA, USA), in 6-well plates at 37°C, in a 100% humidity cell incubator containing 5% CO_2_, and identified with human anti-factor VIII antibody (Santa Cruz Biotechnology, Inc., Santa Cruz, CA, USA). The cells cultured were harvested for assays during the exponential growth phase. The exponential growth EOMA cells (5×10^4^/well) were seeded in 24-well plates containing various agents for 24 h as follows: Naked siRNA^CD31^-RNAi-mate (siRNA^CD31^ group), FAM-labeled siRNA^CD31^-RNAi-mate (siRNA^CD31^-FAM group), SNC-RNAi-mate (SNC group) and Opti-minimum essential medium (MEM; reduced-serum cell culture medium; Gibco) as a blank control (control group). In brief, the siRNA^CD31^-RNAi-mate transfection procedures were as follows: Firstly, 50 μl Opti-MEM and 20 pmol siRNA^CD31^ (or siRNA^CD31^-FAM, SNC or Opti-MEM) were completely mixed, then 50 μl Opti-MEM diluted with 2 μl RNAi-mate reagent was added and the mixture was kept at room temperature for 5 min. Secondly, the diluted siRNA^CD31^ (or siRNA^CD31^-FAM or SNC) and RNAi-mate reagent were mixed gently to form siRNA-lipoplexes at room temperature for 20 min. Finally, 100 μl complexes involving siRNA^CD31^-RNAi-mate or siRNA^CD31^-FAM-RNAi-mate were respectively added to each well containing the cells and the medium used for transfection, and 100 μl SNC-RNAi-mate and Opti-MEM medium were added respectively to the SNC and control wells. The FAM-fluorescence detection was performed with a confocal microscope (excitation wavelength of 495 nm, emission wavelength of 525 nm; Leica, Mannheim, Germany) after transfection efficiency had been reached for 6 h at 37°C in a CO_2_ incubator. The cell transfection rate was ~80%, and the transfection process continued for 48 h. Assessment of the various specimens were carried out for RT-PCR and western blot analysis, and the MTT assay of the cell proliferation rate was performed as previously described (24). Each assay was performed in triplicate and independently repeated three times. The rabbit anti-PECAM-1 and mouse anti-glyceraldehyde-3-phosphate dehydrogenase (GADPH) antibodies were obtained from Santa Cruz Biotechnology, Inc., and the goat anti-rabbit immunoglobulin G monoclonal antibody was purchased from Maixin Technology Co., Ltd., (Shenzhen, Guangdong, China) for the western blot assay. The primer sequences of PECAM-1 used for amplification in the RT-PCR are listed in Table II. The inhibition rate of EOMA cell proliferation was calculated as follows: Inhibition rate of proliferation (%) = [1 - optical density (OD) experimental wells / OD control wells] × 100. The wells containing Opti-MEM were used as the control.

The human lung adenocarcinoma (HLAC) A549 cell line was obtained from the Chinese Academy of Sciences Type Culture Collection (CASTCC; Shanghai, China) and cultivated according to the CASTCC recommendations. The cultured cells were harvested for treatment *in vivo* during the exponential growth phase.

### Tumor implantation

Male, 4–5-week-old BALB/c nude mice [experiment animal number, SCXK (Hu) 2012-002)], weighing ~20 g, were obtained from Shanghai SLAC Laboratory Animal, Co., Ltd., (Shanghai, China) and housed in a specific pathogen-free environment. Abdominal skin tumor xenografts of nude mice were established by subcutaneous injection of 200 μl phosphate-buffered saline [PBS; 13 mM NaCl, 2.7 mM KCl, 1.5 mM KH_2_PO_4_ and 8 mM K_2_HPO_4_ (pH 7.2)] containing a total of 2×10^5^ exponential growth HLAC cells (1×10^5^/100 μl). All animal manipulations were performed in accordance with the National Institutes of Health Guide for the Care and Use of Laboratory Animals, and were approved by the Wenzhou Medical University Animal Care and Use Committee (Wenzhou, Zhejiang, China).

### Delivery of siRNA^CD31^-RNAi-mate lipoplexes in tumor-bearing nude mice

For *in vivo* delivery, the treatments were initiated when the tumor xenografts reached ~85 mm^3^ (day 0). siRNA^CD31^-RNAi-mate complexes were created by administering siRNA-lipoplexes intravenously through single tail vein injections of the total 100 μl solution involving 50 μl (20 μM) siRNA^CD31^, 10 μl RNAi-mate (1 mg/ml) and 40 μl PBS, while the control mice underwent a 100 μl saline injection. Each nude mouse underwent vein injection every other day, a total of five times. The tumor xenograft volumes were measured on days 0, 2, 4, 6 and 10 and were calculated according to the following formula: V = (W^2^ × L)/2, where V is the tumor volume, W is the width and L is the length. The mice were sacrificed by cervical dislocation following the last measurement of the tumor xenograft volumes on day 10. The two divided half-tissues of the tumor xenograft and the lung, brain, liver, heart and kidney tissues were kept in PBS at −80°C for PECAM-1 (Santa Cruz Biotechnology, Inc.) and VEGF (Boster Biological Technology Co., Ltd., Wuhan, China) ELISA examination, respectively, and fixed with formalin for paraffin-embedded tissue sections for PECAM-1 immunohistochemical examination.

### ELISA estimations for PECAM-1 and VEGF

The extract of the homogenate from the tumor xenografts and the lung, brain, liver, heart and kidney tissues was used for measuring the protein concentrations of PECAM-1 and VEGF according to the ELISA kit instructions. Subsequent to stopping the reaction, the plates were read on a KHB-ST-360 microplate reader purchased from Jingong Industrial Co., Ltd., (Shaoxing, Zhejiang, China). The PECAM-1 ELISA kit was obtained from Abgent (San Diego, CA, USA), and the VEGF ELISA kit was purchased from EIAab (Wuhan, Hubei, China). The bicinchoninic acid (BCA; Beyotime Institute of Biotechnology, Shanghai, China) determination of total protein homogenate was used for correcting the value of PECAM-1 and VEGF, and the BCA correction values of PECAM-1 or VEGF in the homogenates were calculated as follows: BCA correction value = measured value / BCA value.

### Statistical analysis

Each assay was performed in triplicate and was independently repeated three times. Results are expressed as the mean ± standard deviation. The difference between two group means was tested by one-way analysis of variance or Student’s t-test according to the character of the experimental data. P<0.05 was considered to indicate a statistically significant difference. All data were processed by SPSS version 16.0 for Windows (SPSS, Inc., Chicago, IL, USA).

## Results

### siRNA^CD31^ is transfected effectively into EOMA cells in vitro

**Figure 1:**
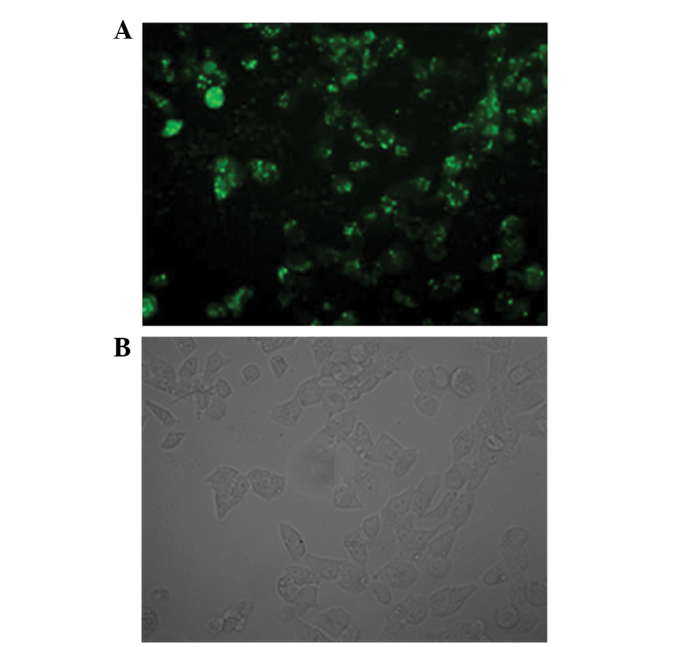
siRNA^CD31^-RNAi-mate complexes successfully transfected into EOMA cells. (A) Bright fluorescence emitted from the EOMA cells transfected by the FAM-labeled siRNA^CD31^. (B) The same EOMA cells observed by optical microscope. siRNA, small interfering RNA; CD, cluster of differentiation; EOMA, murine hemangioendothelioma.

The results of FAM-fluorescence detection by confocal fluorescence microscopy showed that siRNA^CD31^, with RNAi-mate as a carrier, was successfully transfected into the EOMA cells. The bright fluorescence was emitted from the EOMA cells transfected by the fluorescence FAM-labeled siRNA^CD31^ (Fig. 1A). Fig. 1B shows the same cells observed by optical microscopy (Olympus BX-51; Olympus Tokyo, Japan).

### In vitro siRNA^CD31^ inhibits the proliferation of EOMA cells

**Figure 2:**
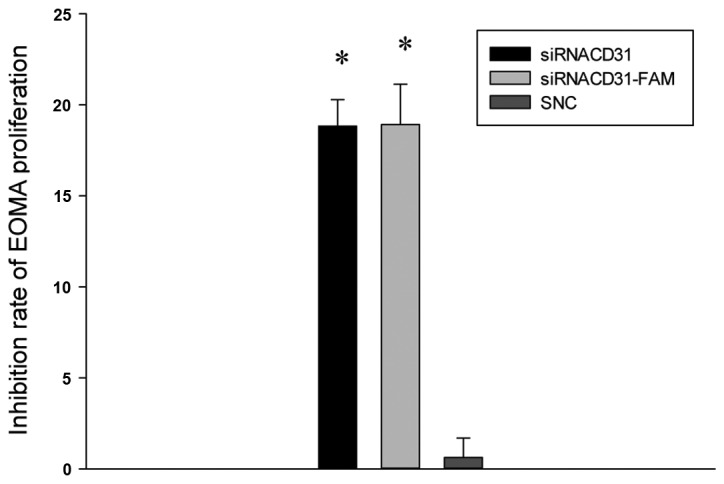
Results of the MTT assay for the inhibition rates of EOMA proliferation showing that siRNA^CD31^ and siRNA^CD31^-FAM effectively inhibit the proliferation of EOMA cells using RNAi-mate as a carrier (all P<0.01 vs. SNC). The inhibition rates of EOMA proliferation are not impaired for the fluorescence FAM-labeled siRNA^CD31^ in the EOMA cells treated by FAM-labeled siRNA^CD31^-FAM-RNAi-mate (i.e., the siRNA^CD31^-FAM group) (P>0.05 vs. siRNA^CD31^). The wells containing Opti-MEM were used as control wells. Each assay was performed in triplicate and was independently repeated three times. The inhibition rate of EOMA proliferation was calculated with the formula: Inhibition rate of proliferation (%) = (1 - OD experimental wells / OD of control wells) × 100. siRNA, small interfering RNA; CD31, cluster of differentiation 31; PECAM-1, platelet endothelial cell molecule 1; EOMA, murine hemangioendothelioma; RNAi, RNA interference; SNC, stable negative control; MEM, mimimum essential medium; OD, optical density. ^*^P<0.01 vs. SNC.

The results of the MTT assay for the inhibition rates of EOMA proliferation showed that the inhibition rates of the siRNA^CD31^ and siRNA^CD31^-FAM groups increased compared with those of the SNC group (all P<0.01 vs. SNC; Fig. 2), and the inhibition rates of EOMA proliferation were not different between the siRNA^CD31^ and siRNA^CD31^-FAM groups (P>0.05; Fig. 2). These results indicate that siRNA^CD31^ inhibited the proliferation of the EOMA cells, and that the fluorescence label, FAM, did not impair the transfection rate of the siRNA^CD31^.

### In vitro siRNA^CD31^ downregulates PECAM-1 mRNA and protein expression

**Figure 3:**
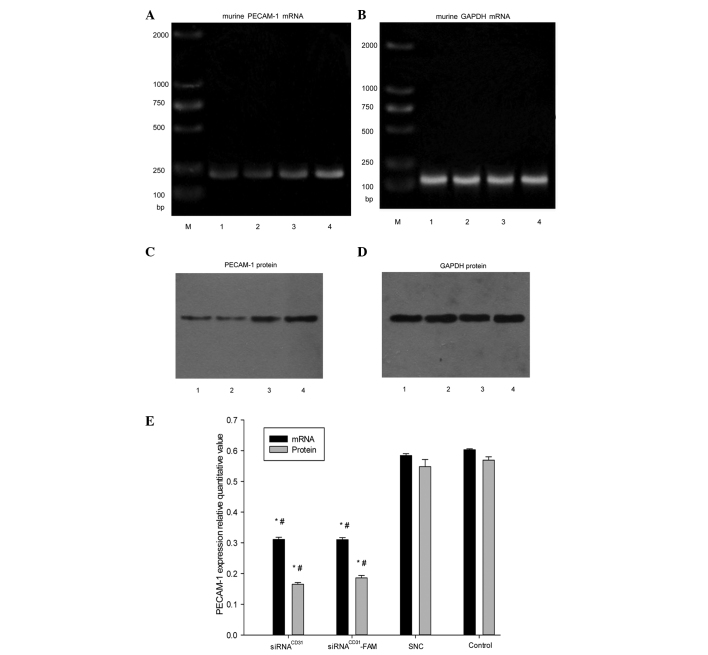
*In vitro* siRNA^CD31^ downregulates the expression of PECAM-1 mRNA and protein in EOMA cells. RT-PCR analysis of (A) PECAM-1 mRNA and (B) GADPH mRNA expression was used as an internal control. Western blot analysis of (C) PECAM-1 protein and (D) GADPH protein was used as an internal control. (E) Bar diagram showing the relative quantitative values of PECAM-1 mRNA and protein determined with RT-PCR and western blot analysis, respectively. siRNA^CD31^ and siRNA^CD31^-FAM, with RNAi-mate as a carrier, downregulated the expression of PECAM-1 mRNA and protein compared with EOMA cells treated with SNC and Opti-MEM (i.e., control group) (all P<0.01 vs. SNC or control) and the effects were not weakened for fluorescence FAM-labeled siRNA^CD31^ (P>0.05, siRNA^CD31^ vs. siRNA^CD31^-FAM). There was no significant difference between the SNC and control groups (P>0.05). The relative quantification value (RQ) of PECAM-1 mRNA (protein) was calculated according to the following equation: RQ PECAM-1 RNA (protein) = IOD PECAM-1 mRNA (protein) / IOD GADPH mRNA (protein). The RT-PCR and western blot analysis data shown were obtained from assays performed in triplicate and independently repeated three times. siRNA, small interfering RNA; PECAM-1, platelet endothelial adhesion molecule 1; CD31, cluster of differentiation 31; EOMA, murine hemangioendothelioma; RT-PCR, reverse transcription polymerase chain reaction; RNAi, RNA interference; SNC, stable negative control; MEM, mimimum essential medium; GAPDH, glyceraldehyde-3-phosphate dehydrogenase; IOD, integrated optical density. M, marker; lane 1, siRNA^CD31^; lane 2, siRNA^CD31^-FAM; lane 3, SNC; lane 4, Opti-MEM used as control. Error bars shows the standard error of the mean. ^*^P<0.01 vs. control; ^#^P<0.01 vs. SNC.

The use of *in vitro* siRNA^CD31^ and siRNA^CD31^-FAM, with RNAi-mate as a carrier, weakened the expression of the PECAM-1 mRNA (Fig. 3A, B and E) and protein (Fig. 3C, D and E) compared with the EOMA cells treated by SNC and Opti-MEM (i.e., control groups) (all P<0.01 vs. SNC or control), and the effects were not weakened for the fluorescence FAM-labeled siRNA^CD31^ (Fig. 3A–E) (P>0.05, siRNA vs. siRNA^CD31^-FAM). There was no difference between the SNC and control groups (P>0.05). The results indicated that siRNA^CD31^ and siRNA^CD31^-FAM downregulated the expression of PECAM-1 mRNA and protein *in vitro*. The transfection efficiency of siRNA^CD31^ was not weakened by the fluorescence label, FAM.

**Figure 4:**
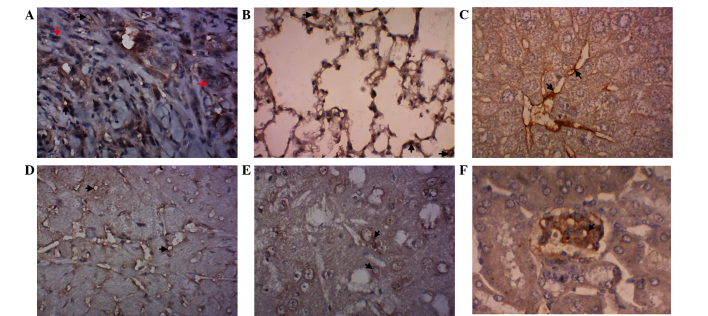
PECAM-1 expression in the vasculature of the (A) lung adenocarcinoma xenograft, (B) lung, (C) liver, (D) heart, (E) brain and (F) kidney, as observed by immunohistochemical analysis. The black arrows show the expression of PECAM-1 in the vascular vessels of various tissues. The red arrow shows the tumor cells. The mice were sacrificed following the measurement of the tumor xenograft volumes on day 10. The tissue specimens were fixed with formalin for paraffin-embedded tissue sections prior to immunohistochemical analysis. PECAM-1, platelet endothelial cell molecule 1.

The expression of PECAM-1 was observed in the vasculature of various tissues. The results of the immunohistochemical examination indicated that PECAM-1 expression was observed in the vasculature of the lung adenocarcinoma xenograft and the lung, liver, heart, brain and kidney tissues (Fig. 4A–F).

### In vivo siRNA^CD31^ inhibits tumor growth

**Figure 5:**
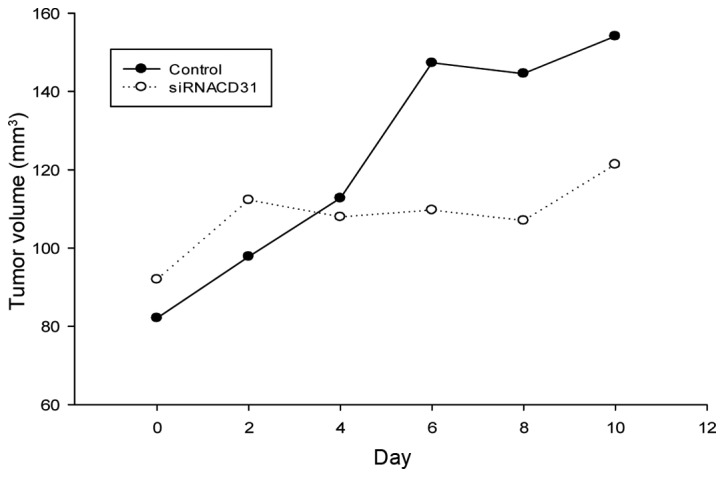
Growth of tumor xenografts of nude mice treated by the tail vein injection of siRNA^CD31^-RNAi-mate complexes (siRNA^CD31^ group) is slower than those treated with saline (i.e., control). The tumor xenograft volumes in the nude mice treated with siRNA^CD31^-RNAi-mate complexes were smaller than those in the control nude mice from day 4, and the deviation of tumor volumes (DV) increased, continuing to day 10. siRNA, small interfering RNA; CD31, cluster of differentiation 31; RNAi, RNA interference.

The volumes of the tumor xenografts in the nude mice treated by siRNA^CD31^, with RNAi-mate (the siRNA^CD31^ group) as the carrier, were smaller than those in the control nude mice (Table III) (P_day 10_<0.05 vs. control; P_deviation of tumor xenograft volume (DV)_<0.01 vs. control). The growth of the tumor xenograft in the nude mice of the siRNA^CD31^ group was slower than in the control group from day 4, and the DV increased in the latter days (Fig. 5). These results indicated that siRNA^CD31^, with RNAi-mate as a carrier, may effectively inhibit the growth of lung adenocarcinoma *in vivo*.

### In vivo siRNA^CD31^ downregulates PECAM-1 and VEGF expression in tumor xenografts

**Figure 6:**
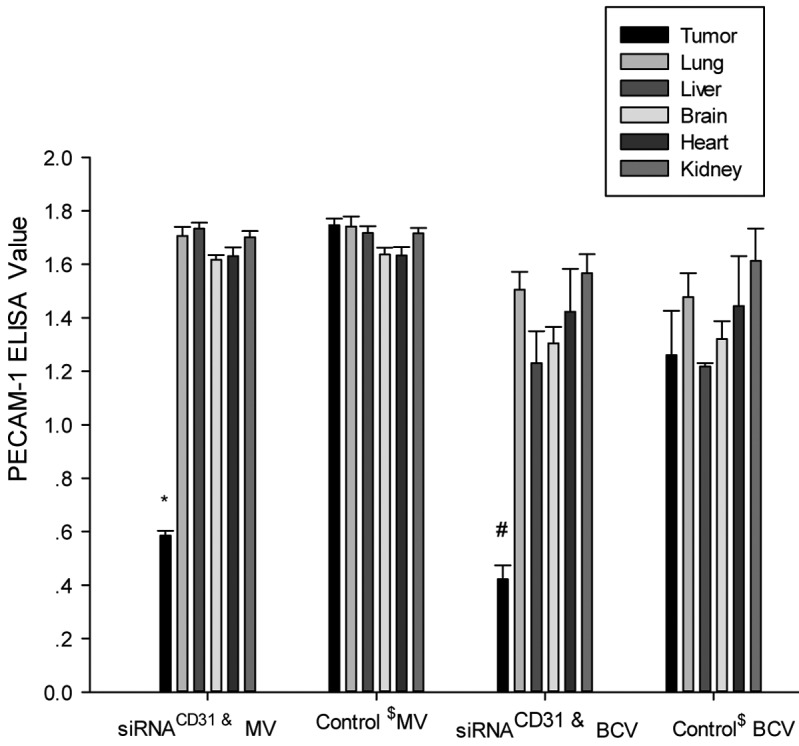
siRNA^CD31^ downregulates PECAM-1 protein expression of tumor xenografts using RNAi-mate as a carrier. The measured values (MV, μg/ml) and BCA correction values (BCV, μg/mg) of PECAM-1 in the tumor xenografts of the nude mice treated with siRNA^CD31^-RNAi-mate complexes (siRNA^CD31^ group) decreased compared with those of the tumor xenograft of the control nude mice treated with saline (control group) (all P<0.01). The MV and BCV of PECAM-1 in other tissues (lung, liver, brain, heart and kidney) of the siRNA^CD31^ group were not different compared with the values of the control group (all P>0.05). Each assay was performed in triplicate and was independently repeated three times. Error bars show the standard error, n=6; ^*^P_MV_<0.01 vs. control MV; ^#^P_BCV_<0.01 vs. control BCV; ^&^siRNA^CD31^ group, the nude mice treated with an injection of siRNA-RNAi-mate via the tail-vein; ^$^control group, the nude mice treated with an injection of saline via the tail-vein; siRNA, small interfering RNA; PECAM-1, platelet endothelial adhesion molecule 1; CD31, cluster of differentiation 31; RNAi, RNA interference; BCA, bicinchoninic acid, for the determination of total protein in homogenate (BCA correction value was calculated by: BCA correction value = measured value / BCA value).

**Figure 7:**
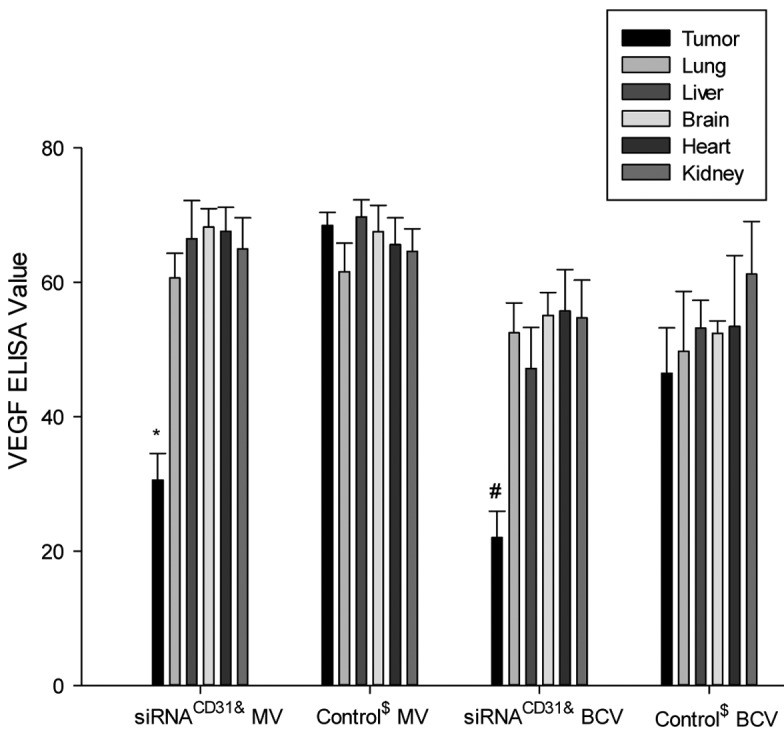
siRNA^CD31^ weakens the VEGF protein expression of tumor xenografts using RNAi-mate as carrier. Measured values (MV, ng/ml) and BCA correction values (BCV, ng/mg) of VEGF in the tumor xenografts of the nude mice treated with siRNA^CD31^-RNAi-mate complexes (siRNA^CD31^ group) decreased compared with those of the tumor xenografts of the control nude mice treated with saline (group control) (all P<0.01). The MV and BCV of VEGF in the other tissues (lung, liver, brain, heart and kidney) of the siRNA^CD31^ group were not different compared with the values of the control group (all P>0.05). Each assay was performed in triplicate and was independently repeated three times. Error bars show the standard error, n=6; ^*^P_MV_<0.01 vs. control MV; ^#^P_BCV_<0.01 vs. control BCV; ^&^siRNA^CD31^ group, the nude mice treated with an injection of siRNA-RNAi-mate via the tail vein; ^$^control group, the nude mice treated with an injection of saline via the tail vein; siRNA, small interfering RNA; CD31, cluster of differentiation 31; VEGF, vascular endothelial growth factor; RNAi, RNA interference; BCA, bicinchoninic acid, for the determination of total protein in homogenate (BCA correction value was calculated by: BCA correction value = measure value / BCA value).

The results of the PECAM-1 protein expression analysis with ELISA indicated that the measured values (MVs) and BCA correction values of PECAM-1 in the tumor xenografts of the nude mice treated with siRNA^CD31^-RNAi-mate complexes (the siRNA^CD31^ group) were decreased compared with the values of the tumor xenografts of the nude mice treated with saline (control group) (all P<0.01; Fig. 6). However, the MVs and BCA correction values of PECAM-1 of the other tissues (lung, liver, brain, heart and kidney) in the nude mice treated with siRNA^CD31^-RNAi-mate complexes were not significantly different compared with the values from the control nude mice treated with saline (all P>0.05; Fig. 6). The VEGF ELISA assay achieved similar results to those of PECAM-1 (Fig. 7).

## Discussion

RNAi is a promising therapeutic approach to silencing disease-causing genes, and is usually mediated by siRNA consisting of 19- to 23-nucleotide double-stranded RNA duplexes. The delivery system is the main complication in achieving gene silencing by siRNA technologies *in vivo*. Previous studies have reported that 2′-O-methyl-modified siRNA possess a strong resistance to the degradation by nuclease in the serum and tissues (20,23). The administration of cationic lipids has been applied for siRNA delivery *in vivo* (22). The endothelial specific marker, PECAM-1 (i.e., CD31) (10,12,26), is closely associated with angiogenesis (27), and the vasculature of the TME plays a significant role in the proliferation and invasion of tumor cells. PECAM-1 could be used as a potential therapeutic target on the TME with respect to its activity in the pathogenesis of tumors, including lung cancer (28,29).

In the present study, the targeted delivery of 2′-O-methyl-modified siRNA^CD31^ and cationic liposome RNAi-mate complexes on endothelial PECAM-1 *in vitro* and *in vivo* were investigated. Three important findings were noted in the present study. The first was that the 2′-O-methyl-modified siRNA^CD31^-lipoplexes effectively silenced the target gene, PECAM-1, *in vitro* and *in vivo*. 2′-O-methyl-modified siRNA^CD31^ successfully downregulated the PECAM-1 mRNA and protein expression of the EOMA cells using RNAi-mate as a carrier *in vitro* (Fig. 3), and the expression of PECAM-1 was detected by immunohistochemical examinations in the vasculature of the lung adenocarcinoma xenografts (Fig. 4A) and in the vascular tissues of the lung, liver, heart, brain and kidney *in vivo* (Fig. 4B–F). These results provided a molecular and cellular basis for targeted treatment with 2′-O-methyl-modified siRNA^CD31^ and RNAi-mate complexes *in vivo*. In the *in vivo* study, the growth of the lung adenocarcinoma xenografts was effectively inhibited by injecting the complexes of 2′-O-methyl-modified siRNA and RNAi-mate via the tail veins of the nude mice (Table III and Fig. 5). Although the expression of the PECAM-1 protein in the lung, liver, heart, brain and kidney tissues was not decreased (Fig. 6), a decrease in PECAM-1 expression was obtained in the lung adenocarcinoma xenografts (Fig. 6). These findings indicated that the 2′-O-methyl-modified siRNA-lipoplexes achieved the targeted silencing of the PECAM-1 gene in the vasculature of the lung adenocarcinoma xenografts *in vivo*. The achievement of specific targeted silencing of the PECAM-1 gene in tumor xenografts is possibly due to the strong bioavailability of the siRNA^CD31^-lipoplexes of the neovascular cells in the TME (22,30). Cationic liposome RNAi-mate may act as a candidate carrier for the systemic administration of siRNA^CD31^ to other liposomes (21,30). Lung adenocarcinoma with abundant vasculature is the most common pathological type of lung cancer. With respect to the limit and toxicity of traditional chemotherapy on lung cancer, target medications have created a promising research area for the bio-therapy of lung cancer (1–4). Besides VEGF (4), PECAM-1 would also be a potential target on the vasculature of the TME (14,15,28), as it plays a significant role in angiogenesis (13,27). Although PECAM-1 activity on the modulation of endothelial cells and its effects in tumor angiogenesis are known (14,15), the effects of targeted delivery of PECAM-1 on the growth of lung cancer requires further investigation. It has been demonstrated that 2′-O-methyl-modified siRNA and cationic lipids could be applied as an effective targeted delivery for silencing a target gene (22). On the basis of the previous discussions regarding siRNA delivery systems, the present study possibly provides an important target strategy involving 2′-O-methyl-modified siRNA targeting PECAM-1 using cationic lipids RNAi-mate against the proliferation of lung carcinoma cells (20,22,23).

The second important finding in the present study was that the proliferation of the EOMA cells was inhibited by 2′-O-methyl-modified siRNA^CD31^ using RNAi-mate as a carrier (Fig. 2). PECAM-1 is a membrane protein with signal transduction (10,31,32) occurring via the initiation of downstream signaling pathways, including mitogen-activated protein kinase (33), Erk (34,35) and PI-3/Akt (36). This has significant implications in the regulation of endothelial apoptosis (37,38). Therefore, it is speculated that this finding possibly contributes to the initiation of signal transduction by PECAM-1 and to the induction of EOMA cell apoptosis, leading to the inhibition of the proliferation of EOMA cells by 2′-O-methyl-modified siRNA^CD31^.

The third significant finding was that a simultaneous decrease in PECAM-1 (Fig. 6) and VEGF (Fig. 7) was observed when 2′-O-methyl-modified siRNA^CD31^ downregulated PECAM-1 expression. It is well known that PECAM-1 initiates signal transduction to activate the downstream signaling pathway (31,33–36,39) and regulate the generation and release of bio-mediators (27,32,40,41). On the basis of the aforementioned studies and the results of the present study, we believe that the downregulation of PECAM-1 expression using siRNA^CD31^ silencing of the PECAM-1 gene possibly executed an effect on the signal transduction of PECAM-1, leading to the decrease in VEGF protein expression.

In summary, the present study demonstrated that 2′-O-methyl-modified siRNA^CD31^ and RNAi-mate complexes may effectively silence the PECAM-1 gene *in vitro* and *in vivo*, and downregulate the expression of PECAM-1 and VEGF proteins. siRNA^CD31^ targeting of PECAM-1 in the TME may be a potential gene therapy for tumors. PECAM-1 regulated the generation of VEGF possibly through the signaling pathway involving PECAM-1. However, the improvement of the delivery system of siRNA^CD31^ for achieving complete dissolution of the tumor xenografts with siRNA^CD31^ mediated by lipoplexes and the regulation mechanism of PECAM-1 on VEGF requires further exploration. The combination of various cytokines, including PECAM-1, VEGF, transforming GF and fibroblast GF, contributing to tumor angiogenesis would be a possible candidate for future study.

## Figures and Tables

**Table I:** siRNA sequence for EOMA cells.

siRNA	Sequence (5′ to 3′)
CD31	
Sense	CAGAUACUCUAGAACGGAA
Antisense	UUCCGUUCUAGAGUAUCUG
CD31-FAM	
Sense	CAGAUACUCUAGAACGGAA
Antisense	UUCCGUUCUAGAGUAUCUG-FAM
SNC	
Sense	UUCUCCGAACGUGUCACGUTT
Antisense	ACGUGACACGUUCGGAGAATT

Underlining of certain bases represents 2′-O-methyl modification. siRNA, small interfering RNA; EOMA, murine hemangioendothelioma; CD, cluster of differentiation; FAM, 3′-fluorescein amidite fluorescence-labeled; SNC, stable negative control RNA.

**Table II:** Primers sequence for PECAM-1 RT-PCR.

Gene	Sequence (5′ to 3′)
PECAM-1 (murine)	
Sense	TCCAGGCCAGCTGCTCCACTT
Antisense	GCCTTCCGTTCTCTTGGTGAGGC
GAPDH (murine)	
Sense	AACTTTGGCATTGTGGAAGG
Antisense	GGATGCAGGGATGATGTTCT

PECAM-1, platelet endothelial adhesion molecule 1; RT-PCR, reverse transcription polymerase chain reaction; GAPDH, glyceraldehyde-3-phosphate dehydrogenase (internal control gene).

**Table III:** Change of tumor xenograft volumes.

Group	V_0_, mm^3^	V_10_, mm^3^	DV, mm^3^
siRNA^CD31^	91.990±6.562	121.346±5.935^a^	29.356±2.917^b^
Control	82.127±17.033	154.082±28.563	71.954±19.938

Mean ± standard deviation, n=6.

a P <0.05 vs. control;

b P<0.01 vs. control.

V_0_, tumor xenograft volume on day 0; V_10_, day 10; DV, deviation of tumor xenograft volume (DV = V10 - V0); siRNA, small interfering RNA; CD31, cluster of differentiation 31; siRNA^CD31^, nude mice treated with a tail vein injection of siRNA^CD31^-RNAi-mate complexes; control, nude mice treated with a tail vein injection of saline.

## References

[1] Kim ST, Uhm JE, Lee J (2012). Randomized phase II study of gefitinib versus erlotinib in patients with advanced non-small cell lung cancer who failed previous chemotherapy. Lung Cancer.

[2] Cohen MH, Williams GA, Sridhara R, Chen G, Pazdur R (2003). FDA drug approval summary: gefitinib (ZD1839) (Iressa) tablets. Oncologist.

[3] Perez-Soler R (2004). The role of erlotinib (Tarceva, OSI 774) in the treatment of non-small cell lung cancer. Clin Cancer Res.

[4] de Gramont A, Van Cutsem E (2005). Investigating the potential of bevacizumab in other indications: metastatic renal cell, non-small cell lung, pancreatic and breast cancer. Oncology.

[5] Park S, DiMaio TA, Scheef EA, Sorenson CM, Sheibani N (2010). PECAM-1 regulates proangiogenic properties of endothelial cells through modulation of cell-cell and cell-matrix interactions. Am J Physiol Cell Physiol.

[6] Woodfin A, Voisin MB, Nourshargh S (2007). PECAM-1: a multi-functional molecule in inflammation and vascular biology. Arterioscler Thromb Vasc Biol.

[7] Bautch VL (2012). VEGF-directed blood vessel patterning: from cells to organism. Cold Spring Harb Perspect Med.

[8] Yang L, Guan H, He J, Zeng L, Yuan Z, Xu M, Zhang W, Wu X, Guan J (2012). VEGF increases the proliferative capacity and eNOS/NO levels of endothelial progenitor cells through the calcineurin/NFAT signalling pathway. Cell Biol Int.

[9] Holash J, Davis S, Papadopoulos N (2002). VEGF-Trap: a VEGF blocker with potent antitumor effects. Proc Natl Acad Sci USA.

[10] Ilan N, Madri JA (2003). PECAM-1: old friend, new partners. Curr Opin Cell Biol.

[11] Watt SM, Gschmeissner SE, Bates PA (1995). PECAM-1: its expression and function as a cell adhesion molecule on hemopoietic and endothelial cells. Leuk Lymphoma.

[12] Müller AM, Hermanns MI, Skrzynski C, Nesslinger M, Müller KM, Kirkpatrick CJ (2002). Expression of the endothelial markers PECAM-1, vWf, and CD34 in vivo and in vitro. Exp Mol Pathol.

[13] DeLisser HM, Christofidou-Solomidou M, Strieter RM, Burdick MD, Robinson CS, Wexler RS, Kerr JS, Garlanda C, Merwin JR, Madri JA, Albelda SM (1997). Involvement of endothelial PECAM-1/CD31 in angiogenesis. Am J Pathol.

[14] Zhou Z, Christofidou-Solomidou M, Garlanda C, DeLisser HM (1999). Antibody against murine PECAM-1 inhibits tumor angiogenesis in mice. Angiogenesis.

[15] Tachezy M, Reichelt U, Melenberg T, Gebauer F, Izbicki JR, Kaifi JT (2010). Angiogenesis index CD105 (endoglin)/CD31 (PECAM-1) as a predictive factor for invasion and proliferation in intraductal papillary mucinous neoplasm (IPMN) of the pancreas. Histol Histopathol.

[16] Herbst RS, Ansari R, Bustin F (2011). Efficacy of bevacizumab plus erlotinib versus erlotinib alone in advanced non-small-cell lung cancer after failure of standard first-line chemotherapy (BeTa): a double-blind, placebo-controlled, phase 3 trial. Lancet.

[17] Prager GW, Lackner EM, Krauth MT (2010). Targeting of VEGF-dependent transendothelial migration of cancer cells by bevacizumab. Mol Oncol.

[18] Inai T, Mancuso M, Hashizume H (2004). Inhibition of vascular endothelial growth factor (VEGF) signaling in cancer causes loss of endothelial fenestrations, regression of tumor vessels, and appearance of basement membrane ghosts. Am J Pathol.

[19] Lagnien-Gaume V, Jehl J, Manzoni P (2011). Bevacizumab and lung cancer: eligible patients in daily practice. Rev Mal Respir.

[20] Soutschek J, Akinc A, Bramlage B (2004). Therapeutic silencing of an endogenous gene by systemic administration of modified siRNAs. Nature.

[21] Chien PY, Wang J, Carbonaro D (2005). Novel cationic cardiolipin analogue-based liposome for efficient DNA and small interfering RNA delivery in vitro and in vivo. Cancer Gene Ther.

[22] Aleku M, Schulz P, Keil O (2008). Atu027, a liposomal small interfering RNA formulation targeting protein kinase N3, inhibits cancer progression. Cancer Res.

[23] Czauderna F, Fechtner M, Dames S (2003). Structural variations and stabilising modifications of synthetic siRNAs in mammalian cells. Nucleic Acids Res.

[24] Ouyang JS, Li YP, Li CY (2012). Mitochondrial ROS-K+ channel signaling pathway regulated secretion of human pulmonary artery endothelial cells. Free Radic Res.

[25] Bidwell GL, Perkins E, Raucher D (2012). A thermally targeted c-Myc inhibitory polypeptide inhibits breast tumor growth. Cancer Lett.

[26] Feng D, Nagy JA, Pyne K, Dvorak HF, Dvorak AM (2004). Ultrastructural localization of platelet endothelial cell adhesion molecule (PECAM-1, CD31) in vascular endothelium. J Histochem Cytochem.

[27] Dimaio TA, Wang S, Huang Q, Scheef EA, Sorenson CM, Sheibani N (2008). Attenuation of retinal vascular development and neovascularization in PECAM-1-deficient mice. Dev Biol.

[28] DeLisser H, Liu Y, Desprez PY (2010). Vascular endothelial platelet endothelial cell adhesion molecule 1 (PECAM-1) regulates advanced metastatic progression. Proc Natl Acad Sci USA.

[29] Delisser HM (2007). Targeting PECAM-1 for anti-cancer therapy. Cancer Biol Ther.

[30] Aleku M, Fisch G, Möpert K, Keil O, Arnold W, Kaufmann J, Santel A (2008). Intracellular localization of lipoplexed siRNA in vascular endothelial cells of different mouse tissues. Microvasc Res.

[31] Newman PJ, Newman DK (2003). Signal transduction pathways mediated by PECAM-1: new roles for an old molecule in platelet and vascular cell biology. Arterioscler Thromb Vasc Biol.

[32] Ilan N, Mahooti S, Rimm DL, Madri JA (1999). PECAM-1 (CD31) functions as a reservoir for and a modulator of tyrosine-phosphorylated beta-catenin. J Cell Sci.

[33] Wang Y, Sheibani N (2006). PECAM-1 isoform-specific activation of MAPK/ERKs and small GTPases: implications in inflammation and angiogenesis. J Cell Biochem.

[34] Masuda M, Kogata N, Mochizuki N (2004). Crucial roles of PECAM-1 in shear stress sensing of vascular endothelial cells. Nihon Yakurigaku Zasshi.

[35] Fujiwara K, Masuda M, Osawa M, Kano Y, Katoh K (2001). Is PECAM-1 a mechanoresponsive molecule?. Cell Struct Funct.

[36] Limaye V, Li X, Hahn C, Xia P, Berndt MC, Vadas MA, Gamble JR (2005). Sphingosine kinase-1 enhances endothelial cell survival through a PECAM-1-dependent activation of PI-3K/Akt and regulation of Bcl-2 family members. Blood.

[37] Bergom C, Goel R, Paddock C, Gao C, Newman DK, Matsuyama S, Newman PJ (2006). The cell-adhesion and signaling molecule PECAM-1 is a molecular mediator of resistance to genotoxic chemotherapy. Cancer Biol Ther.

[38] Wu N, Kurosu T, Oshikawa G, Nagao T, Miura O (2013). PECAM-1 is involved in BCR/ABL signaling and may downregulate imatinib-induced apoptosis of Philadelphia chromosome-positive leukemia cells. Int J Oncol.

[39] Masuda M, Osawa M, Shigematsu H, Harada N, Fujiwara K (1997). Platelet endothelial cell adhesion molecule-1 is a major SH-PTP2 binding protein in vascular endothelial cells. FEBS Lett.

[40] Enciso JM, Gratzinger D, Camenisch TD, Canosa S, Pinter E, Madri JA (2003). Elevated glucose inhibits VEGF-A-mediated endocardial cushion formation: modulation by PECAM-1 and MMP-2. J Cell Biol.

[41] Privratsky JR, Tilkens SB, Newman DK, Newman PJ (2012). PECAM-1 dampens cytokine levels during LPS-induced endotoxemia by regulating leukocyte trafficking. Life Sci.

